# Outcomes of Cases of Prenatally-Diagnosed Congenital Pulmonary Airway Malformation

**DOI:** 10.1055/s-0039-1697983

**Published:** 2019-11

**Authors:** Mehmet Sinan Beksac, Erdem Fadiloglu, Atakan Tanacan, Canan Unal, Neslihan Bayramoglu Tepe, Emine Aydın, Gokcen Orgul, Murat Yurdakok

**Affiliations:** 1 Division of Perinatology, Department of Obstetrics and Gynecology, Hacettepe University, Ankara, Turkey; 2 Division of Neonatology, Department of Pediatrics, Hacettepe University, Ankara, Turkey

**Keywords:** prenatal diagnosis, congenital pulmonary airway malformation, congenital lung masses

## Abstract

**Objective** To evaluate the outcomes of cases of prenatally-diagnosed congenital pulmonary airway malformation (CPAM).

**Methods** We retrospectively evaluated cases of prenatally-diagnosed CPAM between 2004 and 2018. Ultrasonographic features such as visualization of a fetal lung mass and heterogeneous pulmonary parenchyma were used for CPAM diagnosis. Prenatal and postnatal findings were compared in terms of accuracy regarding the CPAM diagnosis.

**Results** The sample consisted of 27 cases. There were four cases in which the patients opted for the termination of pregnancy due to the severity of the lesion. A total of 23 neonates were delivered, and CPAM was confirmed in 15 cases. The median gestational age at delivery was 37 weeks (28–40 weeks) and the mean birth weight was 2,776 g. There were two neonatal deaths, one due to pneumothorax, and the other due to hypoplastic left heart syndrome (HLHS). A total of five patients with respiratory problems were operated in the postpartum period. There were eight misdiagnosis: bronchopulmonary sequestration (five cases), congenital lobar emphysema (two cases), and congenital diaphragm hernia (one case).

**Conclusion** A precise postnatal diagnosis is very important to organize the proper management of the pregnancies with fetuses with CPAM. The positive predictive value of the prenatal diagnosis of CPAM via ultrasonography is of 70.3%. The differential diagnosis of CPAM may be prolonged to the postpartum period in some cases.

## Introduction

Congenital pulmonary airway malformations (CPAMs), formerly known as congenital cystic adenomatoid malformation (CCAM), congenital lobar hyperinfilation (CLH), bronchopulmonary sequestration (BPS), and bronchogenic cyst (BC), are the main congenital lung malformations (CLMs) detected prenatally.^1^ ^2^ ^3^ The prenatal diagnosis of these malformations is critical for the proper management of patients in the prenatal and postnatal periods, including management options such as deliveries of patients at a tertiary center and choice for termination of pregnancy in necessary cases.

The underlying pathophysiological mechanisms are not clear, but bronchopulmonary developmental deficiencies probably occur at the early stages of embryogenesis, which is responsible for these lung masses.^4^ Congenital pulmonary airway malformation is considered a hamartomotous lesion of the bronchogenic tree or an arrest in the development of the bronchial tree.^5^ Most prenatally-detected cases of CPAM progress until the 28th gestational week and start to regress by this time.^6^ ^7^ A serial fetal ultrasonography is mandatory to determine lesion growth and to detect potential complications as soon as possible. Moreover, the postnatal examination of these newborns using various imaging methods (especially computed tomography) is necessary for their future managements (expectant or surgery) after delivery.^8^

Congenital pulmonary airway malformation is not a common disease, with a reported incidence of 0.94 per 10,000 live births, and with predominance of large cystic subtypes.^6^ The disease results from the malformation of the bronchopulmonary tree at different levels, and presents with a fetal lung mass. The diagnosis is possible with a suspected fetal ultrasonography. A careful management must be performed for a favorable pregnancy outcome and postnatal management, after counseling with the parents.

The early prenatal diagnosis of CPAMs changes the routine pregnancy follow-up, and all interventions must be performed at experienced centers by a team composed of obstetricians, pediatricians, radiologists, pediatric pathologists, pediatric surgeons, and thoracic surgeons. In the present study, we aimed to share our experience with prenatally-detected cases of CPAM.

## Methods

**Fig. 1:**
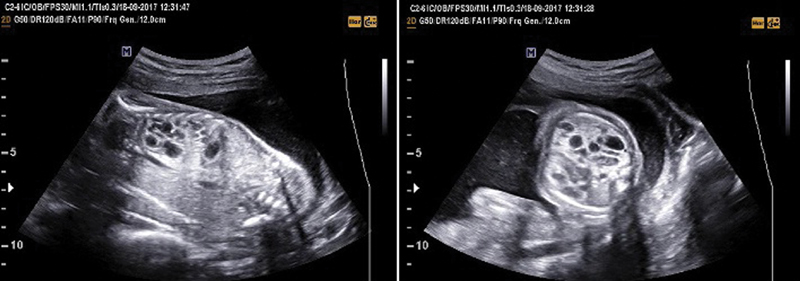
Prenatal ultrasonographic features of a CPAM case in our series.

We retrospectively evaluated cases of prenatally-diagnosed CPAM between 2004 and 2018 at the Division of Perinatal Medicine of Hacettepe University, Ankara, Turkey. Ultrasonographic features such as visualization of a fetal lung cyst/mass and heterogeneous pulmonary parenchyma were used for CPAM diagnosis (Fig. 1). The prenatal diagnosis of CPAM was confirmed by two expert clinicians. We have only included cases with a prenatal diagnosis of CPAM. Doppler sonography was also used for the differential diagnosis of CLMs.

All neonates were evaluated at the Department of Neonatology of our institution for the confirmation of the prenatal diagnosis of CPAM by using further imaging tools (chest X-ray, ultrasonography, and computed tomography or magnetic resonance imaging). In cases in which it was deemed necessary, surgical interventions were performed at our medical center, and all specimens were evaluated histopathologically. Fetal autopsy results were obtained from the Department of Fetal and Adult Pathology for the terminated fetuses.

The Non-Interventional Clinical Research Ethics Board of our institution approved the study under number GO 16/189.

## Results

**Fig. 2:**
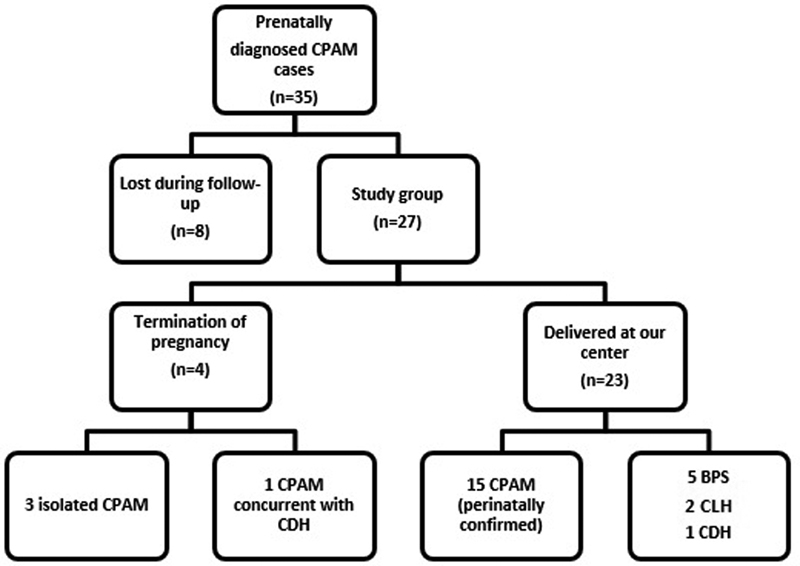
Study design. Abbreviations: BPS, bronchopulmonary sequestration; CDH, congenital diaphragmatic hernia; CLH , congenital lobar hyperinflation; CPAM, congenital pulmonary airway malformation .

There were 35 fetuses with prenatally-diagnosed CPAM during the study period. Cases (*n* = 8) in which patients were delivered at other medical centers or lost during follow-up were excluded from the study because of a lack of sufficient information related to pregnancy outcomes and neonatal period findings. The remaining 27 patients were included in the final stage of the study (Fig. 2). The mean gestational age at the time of the prenatal diagnosis of CPAM was 24 weeks (18–30 weeks).

There were four cases in which the pregnancies were terminated because of the severity of the clinical findings and associated congenital abnormalities. The prenatal diagnosis of CPAM was confirmed in three cases, and the fourth case was reported to have a complex congenital diaphragmatic hernia (CDH), concurrent with CPAM. Early onset of severe mediastinal shift was confirmed in all terminated cases.

The rest of the pregnancies (*n* = 23) were followed up at our center until delivery. All neonates were reevaluated after the delivery, and CPAM was confirmed in 15 cases: 5 cases of BPS, 1 of CDH, and 2 of CLH. In the present study, the prenatal diagnosis of CPAM by ultrasonographic evaluation was confirmed in 70.3% (19 out of 27) of the cases (4 by fetal autopsy and 15 by the evaluation of the neonates in the postpartum period).

The mean maternal age in these 19 cases was 27.5 (19–37) years. We observed that 68.4% (13 out of 19) of the fetuses were male, and 94.7% of the cases were unilateral (the site of the lesion was on the right side in 11 cases, on left side in 7 cases, and bilateral in 1 case). The case with prenatally diagnosed bilateral CPAM was also confirmed at neonatal period during the surgery. The fetal autopsy results and the postpartum evaluation of the neonates revealed that type-1, type-2 and type-3 CPAM were observed in 6 (31.6%), 6 (31.6%) and 7 (36.8%) cases respectively (Table 1).

**Table 1:** Characteristics of the patients with definitive diagnosis of congenital pulmonary airway malformation

Gestational age at the time of the diagnosis (weeks)	24 weeks (18–30 weeks)
Median gestational age at the time of delivery^ǂ^	37 (28–40)
Mean birth weight (grams)^ǂ^	2,776 (1,500–3,940)
Gender of the fetus	
* Male*	13
* Female*	6
Site of the lesion	
* Right*	11
* Left*	7
* Bilateral*	1
Associated anomalies	
* Single umbilical artery, phocomelia*	1
* Hypoplastic left heart syndrome*	1
* Ureteropelvic junction obstruction*	1
Complications	1
* Polyhydramniosis*	3
* Preterm labor preeclampsia*	2
Hystopathology	
* Type-1 CPAM*	6
* Type-2 CPAM*	6
* Type-3 CPAM*	7
Surgery^γ^	6/13 (46.1%)
Postpartum mortality^ǂ^	2/15 (13.3%)
Mean time between delivery and surgery (weeks)	8.8

Abbreviations: CPAM, congenital pulmonary airway malformation; TOP, terminaton of pregnancy.

Notes: ^ǂ^TOP cases were excluded; ^γ^TOP cases and neonatal mortalities were excluded.

Regarding the CPAM-only cases, the 15 mothers delivered at our medical center and were evaluated separately; they had a median gestational age at delivery of 37 weeks (28–40 weeks), and the newborns had a mean birth weight of 2,776 g (1500–3940g). The preterm delivery rate was of 33% (5/15), and 2 (13.3%) of these deliveries were complicated by preeclampsia. The cesarean section (CS) rate was of 60% (9 out of 15) among patients with fetuses with CPAM.

In 7 out of 15 (46.6%) cases, CPAM was found to be asymptomatic, and surgical intervention (6.6 % case of phocomelia) was not necessary. After six months, this case was lost to follow-up. Respiratory problems were defined in 8 (46.6%) CPAM cases. One (6.6%) patient with CPAM who also had pneumothorax died 6 hours after birth, and another with hypoplastic left heart syndrome (HLHS) died during the neonatal period. The remaining six patients (40%) with respiratory problems were operated on in the postpartum period. All of the patients were submitted to anatomical surgical resections, and there was no residual lesion after the surgery. The mean time interval between delivery and surgery was of 8.8 weeks (1–20 weeks). These six neonates (40%) survived, and were discharged from the hospital in due time.

There were 8 (34.7%) cases with different postnatal diagnoses among the 23 newborns delivered at our center (Table 2): 5 cases diagnosed as BPS, 2 as CLH, and 1 as CDH. One fetus (4.3%) was delivered at the 30th week of gestation, and died after labor because of CDH, together with transposition of the great arteries and dextrocardia. The cases of BPS and CLH were all operated successfully, and the infants were discharged from the hospital. None of the fetuses with BPS had a concurrent CPAM in our series.

**Table 2:** Variables of the cases of misdiagnosis

Definitive dignosis	Gestational week at diagnosis	Suspicious CPAM diagnosis	Birth weight	APGAR score	Surgery
CLH	25w	Type 1	3290	9–10–10	+
CLH	21w 6d	Type 3	3940	9–10–10	+
BPS	34w 4d	Type 2	2580	9–10–10	+
BPS	27w 1d	Type 2	3100	7–9-10	+
BPS	24w 2d	Type 1	3000	9–10–10	+
CDH	22w	Type 1	1500	Postpartum fetal death	−
BPS	23w	Type 1	3350	9–10–10	+
BPS	22w	Type 1	2860	6–8-9	+

Abbreviations: APGAR, appearance, pulse, grimace, activity, respiration; BPS, bronchopulmonary sequestration; CPAM, congenital pulmonary airway malformation; CDH, congenital diaphragmatic hernia; CLH, congenital lobar hyperinflation.

## Discussion

Congenital pulmonary airway malformation is an embryogenesis disorder with an incidence of 0.94 for every 10,000 live births per year.^6^ Although the biological facts behind this congenital malformation are not clear, multiple mechanisms have been proposed to explain CPAMs, such as exaggerated *FGF10* signaling and *HOXB5* gene problems.^4^ ^8^

Even though most fetuses with prenatal diagnosis of CPAM have a good perinatal outcome, serial ultrasonographic examinations and careful management are essential for these pregnancies.^9^ The important complications related to CPAM are polyhydramnios, compression of thoracic structures, mediastinal shift, and hydrops fetalis. Clinicians must consider these potential events and focus on the early detection of such complications to prevent unfavorable pregnancy outcomes.^8^ ^10^ Among fetuses born with CPAM, there was one case of spontaneous pneumothorax and one (6.6%) case of polyhydramniosis as additional complications. In addition, in three cases (6.6%), the patients had an early onset of severe mediastinal shift, and the pregnancies were terminated after counseling with the parents. Our findings support those of previous studies, that CPAM and its related complications must be considered carefully during prenatal screening.^11^ ^12^ Termination of pregnancy may also be the choice of management of patients with severe cases after detailed counseling with the families.^13^ Development of fetal hydrops and existence of larger cysts were shown to be the indicators of poor prognosis.^14^

A fetal karyotype together with a detailed anatomy scan and fetal echocardiography may be considered following the diagnosis of CPAM. However, it has been reported that fetuses with CPAM are commonly euploid.^15^ However, structural abnormalities are frequently concurrent with CPAM, and cardiac defects are the most common one.^16^ The rate of additional malformations in CPAM cases was of 21% (4 out of 19) in the present study. These anomalies (among live-born neonates) were single umbilical artery and phocomelia, HLHS, and ureteropelvic junction obstruction. The fourth abnormality was CDH, and this pregnancy was terminated.

The route of delivery must be decided according to general obstetric indications for pregnancies with fetuses with CPAM.^17^ In the present study, the rate of CSs was of 60% (9/15), and all of them were performed due to obstetric indications. The important point is the potential resuscitation/oxygen need of these neonates after delivery. We have shown that 8 out of 15 (53.3%) live-born babies suffered from respiratory distress, and professional help was required for them in the delivery room. A total of two of these cases died during the postpartum period, and six (40%) of them were submitted to surgery. This finding is similar to the result of a previous study^18^ in which the postnatal symptomatic case rate was of 46%. According to all of these findings, we can conclude that labor must be managed carefully in an experienced tertiary center for proper postpartum management and adequate interventions on the newborn.

According to their histopathological type, CPAMs have been classified into five groups. Type 0 is known as acinar atresia; cases of 1 prominent cyst (> 10 cm) or multiple big cysts (2 to 10 cm) are known as type 1; multiple small cysts (< 2 cm) are considered type 2; large masses with solid component are deemed type 3; and alveolar cysts localized peripherally comprise type 4.^6^ ^8^ ^19^ A definitive postnatal diagnosis of these subgroups is not possible only by means of antenatal ultrasonography; thus, careful evaluation is mandatory for a differential diagnosis.^4^ We observed only 3 histopathological subgroups in our sample: 6 cases of type-1, 6 cases of type-2, and 7 cases of type-3 CPAM.

The postnatal evaluation of the neonates with CPAM is necessary for a definite diagnosis. Computed tomography and X-ray chest screening are commonly used for the precise diagnosis of CPAM.^9^ ^12^ It has been reported that CPAM regresses in ∼ 15% of the cases after birth.^9^ ^11^ In our series, in 7 out of 15 (45.5%) cases, CPAM was found to be asymptomatic and did not necessitate surgical intervention (1 case of phocomelia). In one case of CPAM with pneumothorax, the patient died 6 hours after delivery, and another with HLHS died during the neonatal period. A total of six patients with respiratory problems were submitted to surgery in the postpartum period without long-term complications. The positive predictive value of the ultrasonography in the diagnosis of CPAM was found to be of 70.3% in our series, which may be evaluated as consistent with the previously published literature, which reports positive predictive values ranging between 57% and 90%.^20^ ^21^

The postnatal management of the babies with CPAM depends on the severity of problem-specific clinical symptoms and additional health conditions. Surgery is the only treatment modality in symptomatic cases, and it is recommended to prevent malignant transformation and potential complications such as recurrent pneumonia.^6^ However, carefully selected asymptomatic cases can be followed up, because postnatal spontaneous regression has been reported in the literature.^22^ The optimal time for surgery is 6 to 12 months after birth, but earlier intervention is also possible for symptomatic babies.^23^ In our series, the mean interval between delivery and surgery was of 8.8 weeks (1–20 weeks), which is relatively early when compared with this interval in previously-reported case series.^23^ ^24^

Congenital lung malformations are a wide spectrum of diseases that goes together with bronchopulmonary dilatation. Bronchogenic cyst, BPS, and CLH must be considered during the course of the prenatal diagnosis of CPAM because of their similar ultrasonographic findings.^25^ Most of the cases in the literature with CLH and BC are diagnosed after delivery because of the lack of definitive ultrasonographic features during prenatal examinations.^26^ ^27^ However, the vascularization of BPS is different: it has direct vascular branches originating from the descending aorta. It is not always possible to show the vascular flow in the BPS mass with Doppler velocimetry measurements, so postnatal diagnosis is common in these cases.^12^ ^28^ In our series, there were eight misdiagnoses, which are summarized in Table 2: five cases of BPS, two of CLH, and one case of CDH, whose prenatal ultrasonographic examination results were CPAM. Table 2 shows the pregnancy outcomes of these eight cases. The neonate with CDH died during the early neonatal period. The misdiagnosis of BPS and CLH during the prenatal period did not have a negative effect at the delivery room in our cases. These 7 neonates (5 with BPS and 2 with CLH) were submitted to surgery during the postpartum period successfully without any long-term complications.

## Conclusion

The positive predictive value of the prenatal diagnosis of CPAM is of 70.3%. The differential diagnosis of CPAM may be prolonged after birth in some cases. A careful management strategy must be used for a favorable pregnancy outcome, after counseling with the parents to prevent morbidity and mortality.
